# HEALTH PERSONALITY, HEALTH ACTIVATION, AND GENERAL HEALTH

**DOI:** 10.1093/geroni/igz038.3519

**Published:** 2019-11-08

**Authors:** Nicholas Cone, Angelica Jasper, Peter Martin

**Affiliations:** Iowa State University, Ames, Iowa, United States

## Abstract

The purpose of this investigation was to examine associations between health personality and health activation as predictors of general health. Participants from the study included 3907 individuals 65 years of age and older from AARP® Medicare Supplement Plans insured by UnitedHealthcare Insurance Company. The participants completed a survey including the Health Personality Assessment, the Consumer Health Activation Index, and a single-item assessment of self-rated health. Structural equation modeling determined how health personality predicted health activation, and health activation in turn predicted general health. The hypothesized model fit without direct paths from health personality to general health was not optimal. In a second step, we added direct paths from health openness, health neuroticism, and health conscientiousness to general health. The final model fit was then excellent, x2 (df=2) = 18.26, p < .01, RMSEA = .05, CFI = .99. Health neuroticism and health openness were negatively related to health activation, which indicated participants with high health neuroticism and health openness scores were less health activated. Conversely, higher health agreeableness and conscientiousness were associated with more activation. Pathways from health personality via health activation to general health were tested for mediation. Health activation significantly mediated relationships between health neuroticism, health openness, health agreeableness and health conscientiousness to general health. These findings support health activation accounting for some of the associations between health personality and general health. Health neuroticism, health openness, health agreeableness, and health conscientiousness were more closely connected to health activation than health extraversion.

